# Lambeau de Mac Gregor, solution de sauvetage: à propos d'un cas, clinique et résultat

**DOI:** 10.11604/pamj.2019.33.235.16762

**Published:** 2019-07-19

**Authors:** Mohamed Mahmoud El Hacen, Sid'ahmed Limam, Abdoulay Aw, Kemal Ahmed, Noura Biha, Cheikh Ne

**Affiliations:** 1 Orthopedic Department, Nouakchott Military Hospital, Faculty of Medicine of Nouakchott, Mauritania; 2 Orthopedic Department HCZ, Nouakchott, Mauritania; 3 Orthopedic Department CTGB-CHN, Nouakchott, Mauritania; 4 Rheumatology Department, Nouakchott Military Hospital, Faculty of Medicine of Nouakchott, Mauritania; 5 CTGB-CHN/Faculty of Medicine of Nouakchott, Mauritania

**Keywords:** Lambeau, perte de substance, reconstruction, Flap, loss of substance, reconstruction

## Abstract

Le lambeau inguinal est un lambeau « axial » pédiculé à distance. Il s'agit d'un lambeau très fiable pour les couvertures des grandes pertes de substance du membre supérieur, même si son inconvénient majeur est de nécessiter deux temps opératoires. Il reste un lambeau de choix dans l'arsenal thérapeutique de la chirurgie de la main gauche, en urgence ou en chirurgie réglée. Cela s'explique par son épaisseur graisseuse et son pédicule court, de petit calibre, aux variations anatomiques fréquentes. Le but de cette étude est de montrer son intérêt en mettant en balance ses avantages et ses inconvénients. Nous rapportons le cas d'une patiente âgée de 33 ans qui a présenté suite à un traumatisme ouvert complexe de la main une déformation grave avec fermeture de la première commissure et ankylose de la première articulation métacarpo-phalangienne. Il a été opéré en deux temps avec ouverture la première commissure et arthrodèse M1-trappez stabilisé par embrochage suivi d'une couverture cutané immédiate par un lambeau inguinal de McGregor et en deuxième temps après 21 jours pour sevrage et suture du site donneur. Le résultat anatomique et fonctionnel a été jugé bon. Les patients étaient satisfaits par la cicatrice du site donneur aisément dissimulable dans les sous-vêtements, par la fonctionnalité et l'esthétique de la main. Le lambeau de Mac Gregor est une solution de couverture intéressante avec des avantages non négligeables tant du point de vue fonctionnel qu'esthétique. Sa place devrait être remise en valeur dans l'arsenal thérapeutique des lambeaux de recouvrement des membres.

## Abstract (en)

Inguinal flap is a distant axial pediculated flap. This flap is reliable in covering large losses of substance of the upper limb. Its major drawback is that it requires two-step surgery. Inguinal flap is the elective method in the arsenal of emergency left hand surgery or in common left hand surgery. This is due to its greasy thickness and its short small pedicle, with frequent anatomical variations. The purpose of this study was to show its role imbalance out its advantages and its disadvantages. We report the case of a 33-year female patient presenting with severe deformation with closure of the anterior commissure and first metacarpophalangeal joint ankylosis after complex open trauma of the hand. The patient underwent two-step surgery with opening the first commissure and trapezio-M1 arthrodesis stabilized by plug insertion followed by immediate skin coverage by McGregor's inguinal flap and after 21days by weaning and donor site suture. Anatomical and functional outcome was good. The patient was satisfied with the scar at the donor site, which could be easily covered with clothing, by the functionality and aesthetics of the hand. McGregor's flap is an attractive solution for coverage with significant advantages from a functional and aesthetic point of view. Its role should be enhanced in the therapeutic armamentarium of flaps for limb coverage.

## Introduction

Les pertes de substances cutanées au niveau de la main sont fréquentes dans la pratique quotidienne. Dans certaines situations, la cicatrisation dirigée ou la greffe cutanée n'est plus indiquée, l'intérêt réside dans une autoplastie par un lambeau que ce soit local, locorégional ou à distance. Le lambeau inguinal de Mac Gregor décrit par Mac Gregor et Jackson, n'est qu'une alternative parmi plusieurs. Il s'agit d'un lambeau « axial » pédiculé à distance très fiable pour les couvertures des grandes pertes de substance du membre supérieur, même si son inconvénient majeur est de nécessiter deux temps opératoires. Il reste un lambeau de choix dans l'arsenal thérapeutique de la chirurgie de la main, en urgence ou en chirurgie réglée. Le but de cette étude est de montrer son intérêt en mettant en balance ses avantages et ses inconvénients.

## Patient et observation

### Technique chirurgicale

**Figure 1:**
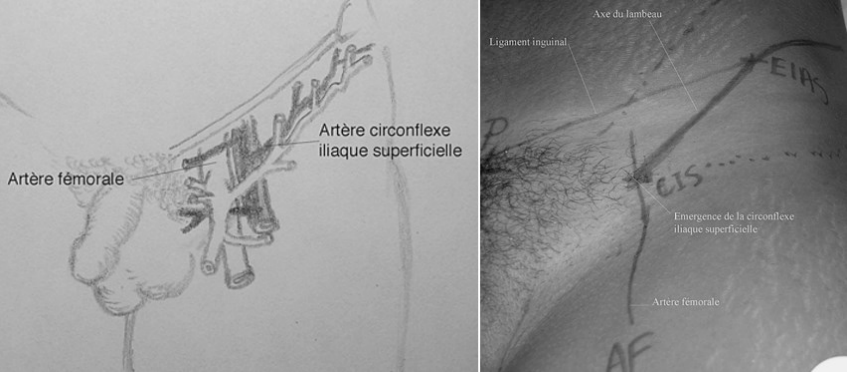
Origine et trajet de l'artère circonflexe iliaque superficielle, branche latérale constante de l'artère fémorale

La taille du lambeau doit être estimée après un parage soigneux de la PDS, et après suppression des rétractions dans le cadre des séquelles de brûlure. Le patient est installé en décubitus dorsal, un billot placé sous la fesse du côté du prélèvement de façon à bien dégager l'aile iliaque. Le champ opératoire incluant à la fois le membre supérieur atteint, la région inguinale et iliaque homolatérale. Le dessin du lambeau est réalisé avant l'installation stérile du champ opératoire. Les repères classiques suivants: 1) l'épine iliaque antéro-supérieure (EIAS); 2) l'épine du pubis; 3) l'arcade crurale (ligne joignant l'EIAS à l'épine du pubis); 4) l'artère fémorale. L'artère circonflexe iliaque superficielle (ACIS), naît de l'artère fémorale 2 à 3cm sous l'arcade crurale. Son trajet suit une ligne rejoignant EIAS (Figure 1). Au-delà de l'EIAS, le lambeau est dessiné à cheval sur la crête iliaque à raison d'un tiers du lambeau au-dessus et deux tiers au-dessous selon le dessin original fait par Mac Gregor. Nous pratiquons une levée du lambeau dont les surfaces supérieure et inférieure sont équidistantes par rapport à la crête. Bien que la tubulisation du lambeau à sa base permet d'éviter les phénomènes de macération et donner à la main une position confortable permettant une rééducation de la main en phase de nourrice. Nous ne la pratiquons pas en raison du risque de plicature du pédicule. Le sevrage est effectué moyennement au 21^ème^jour, après l'épreuve de clampage systématique de 10 minutes.

### Observation

**Figure 2:**
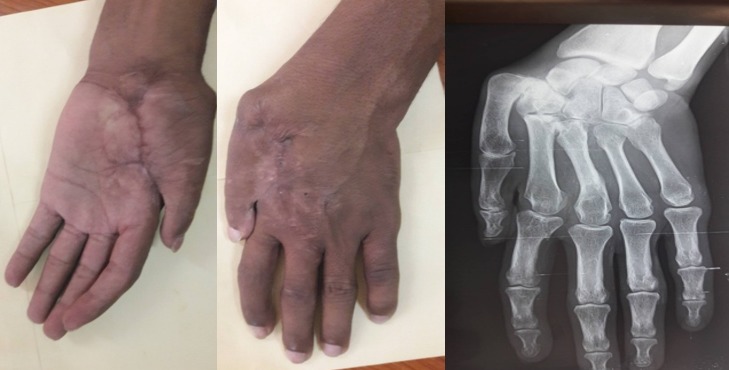
Aspect clinique initial + radiographie initiale

**Figure 3:**
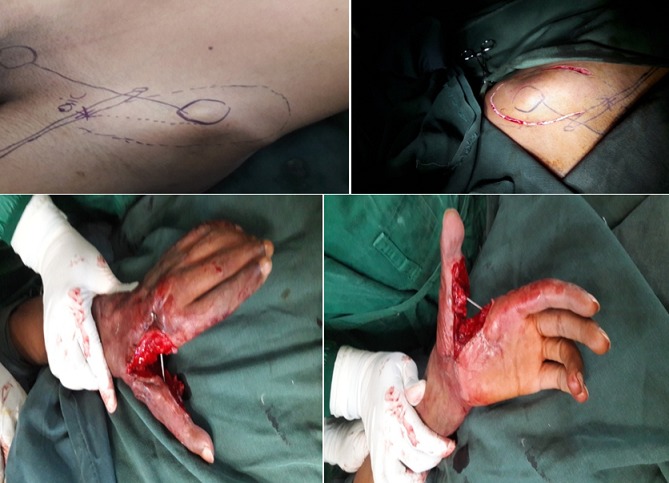
Ouverture de la commissure + traçage du lambeau

**Figure 4:**
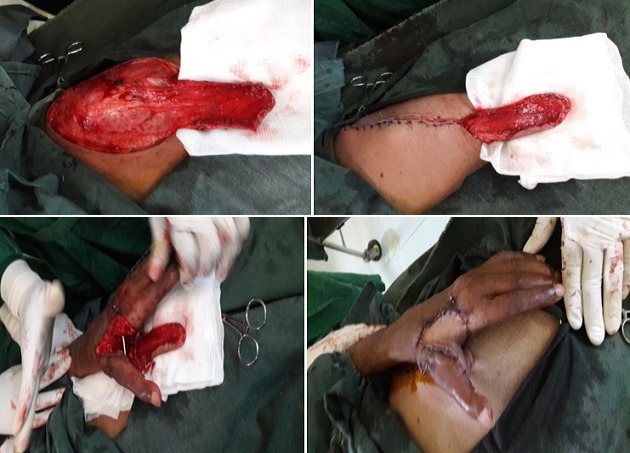
Prélèvement du lambeau et couverture de la commissure

**Figure 5:**
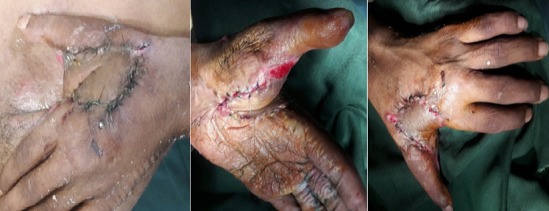
J21 PO: sevrage du lambeau

**Figure 6:**
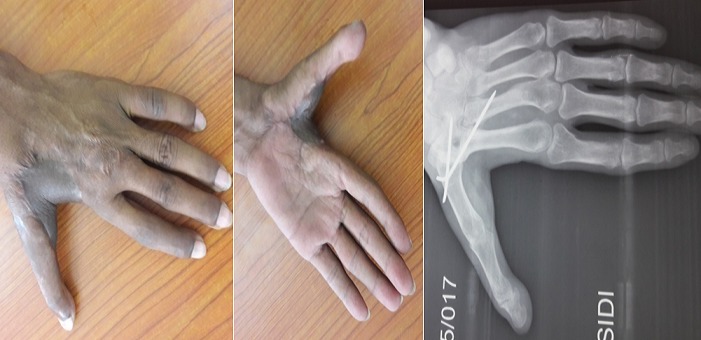
Cicatrisation du lambeau + fusion de l'articulation M1-Trappez

Il s'agit de M. M.W âgée de 33ans qui a consulté pour des séquelles fonctionnelle et esthétique de sa main gauche survenu suite à un traumatisme ouvert complexe da la main mal pris en charge initialement. L'examen initial a objectivé une main gauche d'aspect d'une raquette avec cicatrice de moyenne qualité, fermeture de la 1^ère^ commissure et raideur invincible des chaines digitales rendant la main gauche non fonctionnelle. La radiographie standard a objectivé une luxation invétérée de l'articulation métacarpo-phalangienne du 1^er^ rayon de la main gauche (Figure 2). L'opération avait pour but de corriger le maximum des déformations tant du point de vue fonctionnel qu'esthétique pour retrouver l'ouverture de la 1^ère^ commissure, réduire et stabiliser l‘articulation M1-Trappez. L'opération a été faite sous anesthésie générale, avec installation du membre supérieur gauche et du flanc gauche, le premier temps a consisté à une ouverture de la commissure fixé par une broche M2-M1 qui a laissé une perte substance cutanée impotente, réduction, avivement et stabilisation de l‘articulation M1-Trappez (Figure 3). Dans un second temps nous avons procédé au prélèvement d'un lambeau inguinal de McGregor pour couvrir la 1^ère^ commissure suivi d'un pansement fixant le membre le long du corps qui est maintenu pour trois semaines (Figure 4). Le contrôle postopératoire a montré une vitalité satisfaisante du lambeau avec cicatrisation dans le temps prévu et à J21 il a été repris de nouveau pour sevrage du lambeau (Figure 5). L'évolution a été marquée par la cicatrisation des deux cites opératoires (lambeau et site donneur) et à J45 nous avons enlevé la broche d'ouverture de la commissure âpres la fusion de l'arthrodèse M1-Trappez. Un protocole de kinésithérapie a permis d'améliorer les mobilités des doigts pour plus de fonctionnalité de la main. Le résultat anatomique et fonctionnel a été jugé bon. Les patients étaient satisfaits par la cicatrice du site donneur aisément dissimulable dans les sous-vêtements, par la fonctionnalité et l'esthétique de la main (Figure 6).

## Discussion

McGregor, en 1972 [1], propose ce lambeau, en s'inspirant de la vascularisation du lambeau deltopectoral, centré sur un pédicule artérioveineux propre, en recherchant un lambeau cutané dans une zone de prélèvement dépendant d'un système vasculaire constant, anatomiquement fiable et pouvant être transféré aisément, tout en laissant un préjudice esthétique acceptable. La zone, correspondant à l'artère circonflexe iliaque superficielle, lui paraît le site de prélèvement idéal, permettant de lever un grand lambeau inguinal, avec l'avantage de pouvoir réaliser une tubulisation qui autorise une mobilisation de la main. Le lambeau inguinal est un lambeau « axial » pédiculé à distance. Il s'agit d'un lambeau très fiable pour les couvertures des grandes pertes de substance du membre supérieur, même si son inconvénient majeur est de nécessiter deux temps opératoires. Il reste un lambeau de choix dans l'arsenal thérapeutique de la chirurgie de la main, en urgence ou en chirurgie réglée. La nécrose du lambeau est une complication rare, ne dépassant pas 14% des cas [2] et souvent en zone distale. Elle serait en rapport avec une mauvaise posture du lambeau par plicature du pédicule, ou en cas des lambeaux extrêmes (surface moyenne de 200 cm^2^). Le faible taux de nécrose de ce lambeau confirme sa fiabilité et sa facilité d'exécution [3]. Selon Baron [4], l'infection est d'autant plus fréquente que le lambeau est utilisé en urgence. Le même auteur suggère que la tubulisation du lambeau à sa base permet de réduire les phénomènes de macération et donc l'infection.

L'indication de ce lambeau, dépend de la localisation de la PDS; ainsi au niveau du dos de la main, il prend sa place au dépend des autres lambeaux locorégionaux, principalement lorsque la PDS est assez importante dépassant les 20 cm^2^ de surface [5]. Dans les grandes pertes de substances, nous recommandons comme Tintle *et al.* [6] l'utilisation de lambeaux locorégionaux combines, voire de lambeaux pédicules à distance. Il s'agit en particulier du lambeau inguinal de Mac Gregor permettant de couvrir la main ou l'avant-bras et des lambeaux hétéro-jambier bien que très contraignants gardent des indications en situation précaire [7].

En effet, les lambeaux locorégionaux (le lambeau interosseux postérieur et le lambeau chinois) laissent des cicatrices inesthétiques au site donneur, surtout lorsque la fermeture nécessite une greffe cutanée. Seul le lambeau inguinal de Mac Gregor permet d'obtenir un lambeau auto fermant de grande taille [5]. La PDS cutanée dorsale de la main associée à une PDS osseuse peut être traité par un lambeau composite de Mac Gregor [4]. Le lambeau de Mac Gregor est une solution de couverture intéressante et fiable en cas de *ring finger* qui est un accident grave de pronostic réservé, à la limite de l'amputation. Le lambeau de Mac Gregor garde des avantages multiples, en effet, il s'agit d'un lambeau fiable, facilement et rapidement prélevé, fait de lui une solution de choix de couverture dans les PDS cutanées de la main [8, 9]. Les séquelles esthétiques de cette autoplastie sont minimes [3, 10, 11]. Malgré ces inconvénients: la position déclive et inconfortable de la main durant la phase de nourrice, la nécessité de plusieurs temps opératoires (application, sevrage et dégraissage), et parfois son aspect épais, nous pensons que ce lambeau devrait être connue par les jeunes chirurgiens puisque il constitue parfois une alternative plastique devant l'épuisement des autres.

## Conclusion

Le lambeau inguinal de Mac Gregor est un lambeau « axial » pédiculé à distance très fiable pour les couvertures des grandes pertes de substance du membre supérieur, facile avec un calibre de vaisseaux satisfaisant et peau glabre dans la majorité des cas et rançon cicatricielle minime masquée par les vêtements.

## Conflits d’intérêts

Les auteurs ne déclarent aucun conflit d'intérêts.
